# The Role of Receptor Tyrosine Kinase-like Orphan Receptor 1 (ROR1) in Cancer Stem Cell Signaling

**DOI:** 10.3390/ijms26167828

**Published:** 2025-08-13

**Authors:** Matthew S. Jung, Won-Young Choi, Wenjing Zhang, Francisco N. Barrera, Rachel S. Perkins

**Affiliations:** 1 Department of Pathology, College of Medicine, University of Tennessee Health Science Center, Memphis, TN 38163, USA; mjung6@uthsc.edu (M.S.J.); wchoi11@uthsc.edu (W.-Y.C.); wzhang67@uthsc.edu (W.Z.); 2 College of Graduate Health Sciences, University of Tennessee Health Science Center, Memphis, TN 38163, USA; 3 Center for Cancer Research, College of Medicine, University of Tennessee Health Science Center, Memphis, TN 38163, USA; 4 Department of Biochemistry and Cellular and Molecular Biology, College of Arts and Sciences, University of Tennessee, Knoxville, TN 37916, USA; fbarrera@utk.edu

**Keywords:** ROR1, cancer stem cells, targeted therapy, metastasis, drug resistance

## Abstract

Receptor tyrosine kinase-like orphan receptor 1 (ROR1) is a key regulator of cancer stem cell (CSC) biology and signaling. In CSCs, ROR1 acts as a receptor or co-receptor, interacting with non-canonical WNT ligands, and forming complexes with proteins like CD19 and HER2, to activate diverse downstream signaling pathways. ROR1 signaling in CSCs promotes proliferation, maintains stemness, and enhances migration, invasion, and the epithelial-to-mesenchymal transition (EMT). While minimally expressed after embryogenesis, ROR1 is aberrantly upregulated in numerous cancers, including ovarian, breast, pancreatic, and hematologic malignancies. ROR1 overexpression drives tumor progression, resistance to chemotherapies, disease recurrence, and ultimately metastasis. This expression pattern positions ROR1 as a promising target for CSC-specific therapies. High ROR1 expression is consistently linked to aggressive disease and poor patient outcomes. Here, we review ROR1′s role in CSCs and highlight the complex signaling that is observed in the CSC population. Further, we evaluate the gaps in the current understanding of ROR1 signaling in CSCs and describe how ROR1 regulates the associated signaling pathways. Finally, we provide an up-to-date summary of the promising therapeutic strategies targeting ROR1 that overcome conventional cancer treatment limitations. This review highlights the role of ROR1 as a critical, functional driver of CSCs and adverse patient outcomes across various malignancies.

## 1. Introduction

The landscape of cancer therapy is continuously evolving, driven by an urgent need to improve patient outcomes, and overcome the limitations of conventional treatments. While advancements in surgical techniques, radiation therapy, and chemotherapy have made significant strides, the persistent challenges of drug resistance, metastatic spread, and disease recurrence necessitate the development of novel therapeutic strategies. A growing body of evidence suggests that a subpopulation of cancer cells, known as cancer stem cells (CSCs), plays a critical role in these treatment failures [1,2]. These cells possess unique properties, including the capacity for self-renewal and differentiation, which enable them to initiate and sustain tumor growth, evade therapeutic interventions, and seed new tumors at distant sites [3]. Consequently, understanding the molecular mechanisms that govern CSC behavior is paramount for effective cancer therapies.

Among the various signaling pathways implicated in cancer development and progression, the WNT signaling pathway has emerged as a central player [4]. WNT signaling is crucial for normal embryonic development and tissue homeostasis, and its dysregulation is frequently observed in human malignancies [5]. Within the network of non-canonical WNT signaling, the receptor tyrosine kinase-like orphan receptor 1 (ROR1) has garnered increasing attention due to its restricted expression pattern in normal tissue and its involvement in aggressive cancer phenotypes [6]. ROR1, a type I transmembrane protein, is highly expressed during early embryogenesis and plays a vital role in organogenesis, with its expression significantly diminishing after development in most healthy adult tissues [6,7]. However, ROR1 reappears in a variety of cancers, particularly those characterized by a less differentiated state, suggesting a link to the stemness properties of cancer cells [6,7]. This differential expression profile makes ROR1 a promising target for cancer therapy, potentially allowing for selective targeting of malignant cells while minimizing damage to healthy tissues [8].

**Figure 1:**
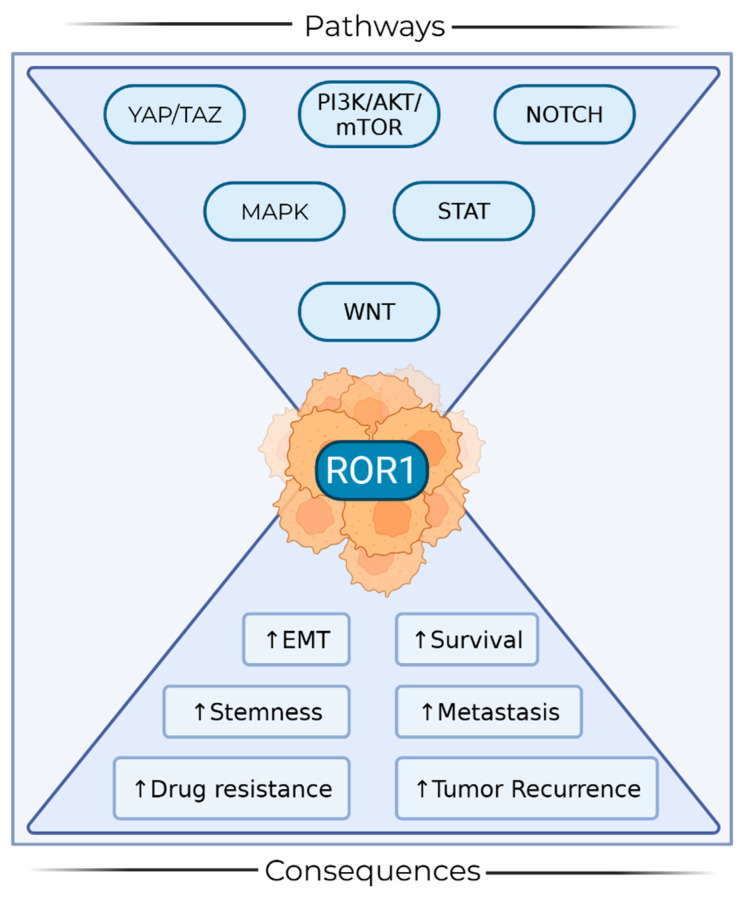
Associated pathways and consequences of ROR1 signaling in CSCs. Graphical summary of ROR1 in CSCs. The top of the funnel depicts signaling pathways associated with ROR1 CSC signaling, and the output of the funnel depicts functional implications of ROR1 crosstalk. ↑ = increase in observance of phenotype. Figure created in BioRender.com.

The importance of ROR1 in CSCs has been observed across multiple cancer types, indicating its involvement in the fundamental characteristics of these cells, such as self-renewal and drug resistance [7,8]. Studies have shown that ROR1 is expressed on CSCs in chronic lymphocytic leukemia, mantle cell lymphoma, glioblastoma, neuroblastoma, osteosarcoma, pancreatic, gastric, fallopian, endometrial, ovarian, and breast cancer, and its inhibition can affect the maintenance of these cells [6,9,10,11,12,13,14,15,16,17,18]. This connection underscores the importance of elucidating the precise role of ROR1 in CSC signaling to facilitate the development of targeted therapies that can effectively eradicate these cells, overcome resistance mechanisms, and ultimately improve patient outcomes. The general role of ROR1 in cancer has been extensively reviewed elsewhere [7,8,19]. However, the objective of this text is to focus on how ROR1 regulates the signaling pathways that govern CSC behavior (Figure 1).

## 2. ROR1 Structure and Function in Development and Cancer

ROR1 and its homolog ROR2 comprise the receptor tyrosine kinase-like orphan receptor (ROR) subfamily of the receptor tyrosine kinase (RTK) family. They are evolutionarily conserved across many domains of life and are essential in early development [20]. The ROR proteins comprise distinct extracellular, transmembrane, and cytoplasmic domains that contribute to their function. RORs are non-canonical WNT receptors that interact with one of ten distinct transmembrane FRIZZLEDs (FZDs) as a co-receptor to direct downstream signaling [19]. The extracellular domain of ROR1 contains an immunoglobulin (Ig)-like domain, a cysteine-rich domain (CRD), and a Kringle domain (KD) [8,20]. The CRD is essential for the binding of WNT ligands, highlighting a direct link between ROR1 and the WNT signaling pathway. The intracellular domain of ROR1 includes a tyrosine kinase domain (TKD) and a proline-rich domain (PRD) [8].

While ROR1 possesses a tyrosine kinase domain, its catalytic activity has been a subject of debate [7]. Evidence has suggested poor catalytic activity, and hence ROR1 might act as a pseudokinase, as its TKD lacks several amino acids or motifs typically required for proper catalytic activity. In vitro studies have indicated that the TKD of ROR1 is unable to bind nucleotides, further supporting its classification as a pseudokinase [21]. Consequently, ROR1 is thought to rely on trans-phosphorylation by other active kinases to initiate downstream signaling events. For instance, ROR1 and ROR2 can undergo tyrosine phosphorylation by kinases such as EGFR, ERBB3, MET, and SRC, leading to the activation of downstream signaling cascades like PI3K-AKT and YAP [22]. Furthermore, a splice variant of ROR1 exists that lacks the tyrosine kinase domain, suggesting potential alternative functions or regulatory mechanisms for the receptor [23]. As a result, it is theorized that ROR1′s kinase activity can be tissue- and disease-specific, as well as co-receptor dependent [7].

The primary function of ROR1 is to act as a receptor or co-receptor for signaling activation by non-canonical WNT ligands: WNT3, WNT5B, WNT16, and most notably WNT5A [8,13,24]. In addition to WNTs, ROR1 has been shown to form a functional complex with CD19 at the cell surface, particularly in the context of mantle cell lymphoma (MCL), leading to the activation of signaling pathways that promote MCL cell proliferation [9]. Moreover, in glioblastoma, the binding of insulin-like growth factor-binding protein 5 (IGFBP5) to ROR1 facilitates the formation of a ROR1/HER2 heterodimer, which subsequently induces the expression of genes like *ETV5* and *FBXW9* to promote glioblastoma stem-like cell invasion and tumorigenesis [25]. This interaction with CD19 and HER2 suggests that ROR1 forms a co-receptor complex to activate signaling pathways not limited to WNT signaling. Additionally, across several tumor types, it has been reported that ROR1 signals with YAP/TAZ to regulate EMT and stemness markers like BMI-1, indicating a common crosstalk between signaling pathways across diverse cancer types [18,26,27]. The ability of ROR1 to bind WNT ligands and crosstalk with other cell signaling molecules underscores its role as a crucial component of cellular signaling networks involved in development and disease progression.

During development, ROR1 signaling is essential for proper regulation of cell polarization, proliferation, and differentiation [28]. On the other hand, ROR1 exhibits aberrant expression in a wide spectrum of human cancers, including both hematological malignancies and solid tumors, and it is frequently associated with aggressive disease states and poor clinical outcomes [9,17]. In fact, ROR1 has been established to have prognostic significance in several cancers, including breast cancer, colorectal cancer, chronic lymphocytic leukemia, ovarian cancer, lung cancer, and diffuse large B-cell lymphoma [17,29,30,31,32,33]. Despite its importance, the mechanism by which ROR1 is reactivated in carcinogenesis is poorly understood, and it is suspected to be disease specific. These questions are subjects of active research. ROR1 promotes cancer cell proliferation, migration, and invasion, which are critical steps in tumor growth and metastasis [34]. The specific roles of ROR1 in cancer can vary depending on the cancer type and the cellular context. However, the association of ROR1 with metastasis and early relapse after therapy further underscores its role in driving tumor aggression [6]. This widespread upregulation of ROR1 across diverse malignancies points towards an important oncogenic role in cancer development and progression and provides insight into its potential as a relevant therapeutic target.

## 3. Cancer Stem Cells

The cancer stem cell theory posits that within a tumor, there exists a distinct subpopulation of cells that possess stem cell-like properties, including self-renewal and the ability to differentiate into the various cell types that constitute the heterogenous tumor [2,35]. These CSCs are believed to be the primary drivers of tumor initiation, progression, metastasis, and recurrence [6,36]. Like normal stem cells, CSCs have the capacity to perpetuate their lineage through self-renewal divisions, ensuring a continuous supply of these tumorigenic cells [37]. The metastatic potential of CSCs is often linked to their ability to undergo EMT [22], their quiescence, and their inherent resistance to known therapies [3]. The presence of CSCs has been verified in many cancers through sphere formation assays, xenografts, and cellular markers of stemness and EMT [3,38], emphasizing the profound relevance of CSCs in cancer biology.

The four primary ways researchers evaluate CSCs are live-cell sorting for surface markers like CD44 or CD34, serial dilutions to isolate subpopulations, Hoescht 33342 dye exclusion, and 3-D tumor spheroids [39]. Spheroids may be the most common and accepted method of isolating CSC populations. Such CSC culturing technique was adapted from normal neural stem cell growth conditions [39]. The method, first adapted in glioblastoma stem cells, cultured cells that were isolated from bulk tumor populations on non-adherent plates in serum-free media supplemented with B27 and N2 (stem cell culture supplements), and fibroblastic and epidermal growth factors [40]. The spheroid protocol originated from the notion that selection of cells that could survive a non-adherent, serum-free environment were those that possessed CSC characteristics [41]. The first glioblastoma stem cell study utilizing this spheroid approach demonstrated that tumor spheroids recapitulated the tumor and expressed stemness markers to significantly higher levels than the standard culture methods [40].

A significant clinical challenge posed by CSCs is their inherent resistance to conventional cancer therapies such as chemotherapy. Unlike the more rapidly dividing bulk tumor cells that are often the primary targets of these treatments, CSCs tend to be slow-cycling or even quiescent, making them less susceptible to the cytotoxic therapies [3]. Moreover, CSCs often express higher levels of drug efflux pumps, possess enhanced DNA repair mechanisms, and exhibit anti-apoptotic tendencies, further contributing to their ability to survive treatment [38]. Consequently, while chemotherapy may effectively shrink the tumor mass by eliminating the more differentiated cancer cells, the residual population of drug-resistant CSCs survives and eventually leads to resistant tumor relapse and recurrence [42]. This problem highlights the critical need for therapies that can specifically target and eradicate CSCs to achieve more durable and curative cancer treatments.

There are many established stemness markers that promote self-renewal, differentiation, metastasis, and drug resistance. Some of the more well-known cancer stemness markers include aldehyde dehydrogenase (ALDH), the CD (cluster of differentiation) family of genes (CD133, CD44, CD117, etc.) and ATP-binding cassette (ABC) transporters (ABCB1, ABCG2, etc.) [43]. The therapeutic challenge is that while these markers are specifically upregulated in CSCs, they are also widely expressed in adult stem cells and normal tissue [44]. Therefore, when considering therapeutic applications in CSC biology, it is essential to identify CSC markers that are either not expressed or very lowly expressed in post-natal tissue. In this manner, ROR1 is a compelling candidate due to its association with poor clinical outcomes [29,32], role in maintaining cancer stemness (Section 4), and low expression in normal tissues [45].

## 4. ROR1 in Cancer Stem Cells

ROR1 expression in CSCs contributes to multiple cancer malignancies. In the following section we summarize the current understanding of ROR1 as a therapeutic target in CSCs for different tumor types.

### 4.1. Gynecological Cancers

Elevated ROR1 expression has been identified as a marker of cancer stemness and EMT in many gynecological malignancies, including fallopian tube epithelial precursor lesions [16], endometrial cancer [46], and epithelial ovarian cancers [26,47]. Clinical data show that an increase in ROR1 expression in ovarian cancers is associated with a higher rate of relapse [17] and decreased overall and disease-free survival rates compared to cancers with low or no ROR1 expression [47]. In addition, elevated ROR1 was significantly associated with increased tumor grade and lymph node metastasis. In vitro, ROR1-positive primary patient-derived xenografts (PDX) express higher levels of the functional stemness marker aldehyde dehydrogenase 1 (ALDH1), have a greater sphere formation efficiency, and induce tumor formation in immunodeficient mice more efficiently than ROR1-negative ovarian cancer cells [47]. ROR1 inhibition via an anti-ROR1 monoclonal antibody or knockdown via shRNA in ovarian PDX decreased sphere formation efficiency and reduced tumor formation in xenografts of virgin mice [47]. Inhibition of ROR1 signaling also decreased invasion of the extracellular matrix and downregulated expression of the EMT marker BMI-1 [47], illustrating the dependency of CSCs on ROR1. Furthermore, ROR1 expression on these ovarian CSCs promotes migration, invasion, and EMT [47].

In addition to stemness, ROR1 is also associated with chemoresistance in ovarian cancer. ROR1-positive tumors display increased treatment resistance compared to ROR1-low or negative tumors [21]. Cisplatin-sensitive and -resistant OVCAR3 spheroids treated with the glucocorticoid dexamethasone showed increased protein levels of ROR1 and its downstream components RhoA, YAP/TAZ, BMI-1, and pAKT [26]. Interestingly, this same result was not observed in OVCAR3 sensitive and resistant cells cultured in traditional, adherent conditions [26], highlighting the CSC-specific ROR1 signaling differences. In the JHOS2 cell line, YAP/TAZ inhibition via verteporfin downregulates ROR1 in both dexamethasone-treated and untreated cells [26]. While not CSC specific, this inhibition highlights a complex crosstalk or feedback loop amongst signaling pathways regulating ROR1 expression. Importantly, inhibiting ROR1 using an anti-ROR1 monoclonal antibody or shRNA increased the efficacy of anti-cancer drugs, suggesting that ROR1 inhibition can assist in overcoming chemoresistance [26]. While these findings shed much-needed light on the role of ROR1 in therapy resistance, the mechanisms of ROR1-induced resistance in CSCs are yet to be determined and present an exciting area for future study.

Given its critical role in resistance, self-renewal, and stemness, ROR1 offers a promising therapeutic target in gynecological cancers. However, there are still gaps in understanding the signaling pathways activated by ROR1 in CSCs that dictate these alterations in stemness, resistance, and metastasis. Understanding the complex role of ROR1 expression and its downstream signaling is critical to the improvement of traditional and combinatorial therapies for patients with gynecological cancers.

### 4.2. Breast Cancer

ROR1 expression is associated with a more aggressive disease progression and increased propensity for metastasis in both HER2+ and HER2− breast cancer [18,48,49]. Additionally, strong ROR1 expression is associated with increased metastasis and is a predictive indicator of overall and disease-free survival in triple-negative breast cancer [33]. In 38 biopsy samples from patients with invasive ductal adenocarcinoma after 3 rounds of chemotherapy, *ROR1*, lncRNA *DLEU2*, markers of CSCs, EMT-related genes, and *BMI-1* were significantly increased [50]. In patient populations with high *DLEU2*, lncRNA-driven *ROR1* expression increased tumor proliferation and chemoresistance, dysregulated cell cycle progression and apoptosis, as well as enhanced metastatic characteristics and sphere formation [50]. The discovery of other regulatory RNAs that govern ROR1 expression is essential to fully understanding the mechanisms that confer stemness. CSCs isolated from patient-derived breast cancer xenografts (ALDH1+/CD44+/CD24Low) had elevated ROR1 levels compared to non-CSC PDX cells. CSCs that expressed high levels of ROR1 displayed significantly greater sphere formation efficiency compared to CSCs with relatively lower expression [18]. ROR1 signaling is also associated with AKT, BMI-1, Rho-GTPase, Hippo-YAP/TAZ [18] and TGFB [50] signaling and a decrease in metastasis-free survival in breast cancer patients [48]. Taken together, these data emphasize ROR1′s role in maintaining stemness, inducing EMT, and promoting metastasis through crosstalk with classic tumorigenic pathways in response to chemotherapy.

In breast cancer, inhibiting ROR1 expression increases sensitivity to chemotherapy, decreases stemness, and inhibits CSCs from forming spheres or engrafting in immunodeficient mice [18,48,50]. Additionally, it was observed that treatment of Hs578T breast cancer with the humanized anti-ROR1 monoclonal antibody cirmtuzumab decreased sphere formation and invasion compared to control in the presence of WNT5A. The combination of cirmtuzumab and paclitaxel was a more effective treatment in mice with breast cancer PDXs than either treatment alone [18]. Additionally, treatment of HER2+ breast cancer cells (patient-derived, HCC1954 and MCF7-HER2+) with the HER2-targeting monoclonal antibody trastuzumab (T-DM1), led to a dramatic increase in ROR1 expression. Inhibition of HER2 and increased levels of ROR1 correlated with the upregulation of YAP/TAZ, which subsequently contributed to treatment resistance to T-DM1 [51]. Conversely, the inhibition of either ROR1 or YAP1 was sufficient to re-sensitize the HER2+ breast cancer cell line HCC1954 to T-DM1 [51]. These findings collectively underscore the potential of ROR1 inhibition as a strategy to overcome CSC-mediated therapy resistance and improve outcomes in breast cancer.

### 4.3. Glioblastoma

ROR1 is overexpressed in glioblastomas and is preferentially expressed within 448 and X01 glioblastoma stem cells (GSCs) compared to total cell populations [25]. ROR1 plays a role in maintaining the stemness of these glioblastoma stem cells, and its expression can be enhanced by signaling pathways active in the CSC niche, such as Notch signaling and hypoxia pathways [34]. These pathways can drive the expression of ROR1 through a WNT5A-ROR1 axis, which in turn promotes the stem cell-like properties and spheroid-forming ability of these cells [34]. Specifically, transcription factors like NICD (Notch intracellular domain) and hypoxia-inducible factor 1 alpha (HIF1α) bind directly to the *ROR1* promoter regions, particularly under spheroid culture conditions that mimic the CSC environment [34].

ROR1 functions by activating several downstream signaling pathways crucial for CSC maintenance and aggressiveness in glioblastoma CSCs. One such pathway involves IGFBP5, which acts as a ligand for ROR1 [25]. This interaction triggers downstream signaling, leading to the phosphorylation of ROR1 itself, as well as HER2 and CREB [25]. Activated CREB then promotes the transcription of genes like *ETV5* and *FBXW9*, which are associated with increased GSC invasion [25]. Inhibition of either ROR1 or HER2 can block this CREB phosphorylation and reduce the invasive capabilities of GSCs [25].

Another critical pathway involves ROR1′s interaction with GRB2, an adaptor protein. ROR1 stabilizes GRB2 by binding, which prevents lysosomal degradation [11]. This ROR1-GRB2 interaction subsequently activates the ERK/c-Fos signaling cascade. The ROR1-GRB2-c-FOS axis is found to be active in glioblastoma patients and correlates with GSC characteristics [11]. The importance of the ROR1-GRB2-c-FOS pathway is underscored by findings that artificial expression of c-Fos reverses the tumor-suppressing effects seen when ROR1 is silenced [11].

ROR1 expression is vital for maintaining key CSC traits. Disrupting ROR1 function significantly impairs GSC proliferation, self-renewal, and the overall growth of glioblastoma tumors in preclinical models [25]. Specifically, knocking down *ROR1* via shRNA in U87MG cells decreased sphere formation, cell invasion and migratory capacity [52]. shRNA inhibition of *ROR1* also decreases mRNA expression of EMT markers, thereby diminishing their stem-like properties and inhibiting metastasis [52]. Therefore, ROR1 is a key driver of CSC characteristics in glioblastoma through cross-signaling networks, making it an attractive therapeutic target to overcome CSC-mediated tumor progression and resistance in glioblastoma.

### 4.4. Pancreatic Ductal Adenocarcinoma

ROR1 heavily contributes to the aggressive nature of pancreatic cancer [14,27]. ROR1 regulates stemness, metastasis, and EMT in pancreatic ductal adenocarcinomas (PDAC) by acting as a downstream target for the methyltransferase *SETD8* [14]. PANC-1 cells transfected with *SETD8* shRNA showed a decrease in ROR1 protein levels, sphere formation, genes related to stemness (such as *SOX2* and *NANOG*) and markers of EMT (like *N-CADHERIN* and *VIMENTIN*), while *E-CADHERIN*, an epithelial marker, increased [14]. The overexpression of ROR1 in these sh*SETD8* cells is sufficient to increase cell migration capacity and protein levels of SOX2, NANOG, and N-CADHERIN [14], validating SETD8 as a regulator of ROR1 expression. Thus, more research understanding the crosstalk between ROR1 and these pathways is critical to our knowledge of PDAC progression and treatment.

CSCs within PDAC exhibit a partial EMT-like state and are characterized by high levels of ROR1 expression [27]. In S2-VP10 PDX, ROR1 promotes proliferation by increasing expression of Aurora kinase B (AURKB) through the activation of the transcription factor E2F via pAKT/C-MYC signaling [27]. Doxycycline-induced inhibition of *ROR1* in the PDAC cell line S2-VP10 decreases overall tumor growth, recurrence after chemotherapy, and metastasis to the lung and mesentery [27]. The transcriptional upregulation of *ROR1* is controlled by the YAP/BRD4 protein complex binding to the enhancer region of *ROR1* [27]. Moreover, the inhibition of the YAP/BRD4 pathway decreases ROR1 expression and subsequently hinders PDAC proliferation and metastasis [27]. Given ROR1′s role in maintaining cancer stemness and metastasis, approaches that effectively neutralize ROR1 activity or its upstream regulators hold significant promise for not only controlling primary tumor growth but also preventing recurrence in pancreatic cancer patients.

### 4.5. Osteosarcoma

In osteosarcoma, ROR1 dimerizes with ROR2 to activate WNT5A/RHOA/DAAM1 signaling in parallel with PI3Ka/AKT to regulate migration and cytoskeletal rearrangement in MG63 and U2OS cells [53]. Furthermore, ROR1 has been shown to express with WNT5B to regulate osteosarcoma stemness in 143B, MG63 and patient-derived xenografts [13]. In vitro, the derivative of Zilovertamab, D10, inhibited sphere formation, increased methotrexate sensitivity and reduced expression of SOX2 demonstrating the role of ROR1 in osteosarcoma CSCs and chemoresistance [13]. Further experiments focusing on the role of additional WNT ligands and the role of ROR1 in maintaining CSCs are an important next step for patients with osteosarcoma.

### 4.6. Hematologic Malignancies

ROR1 is highly expressed on the surface of chronic lymphocytic leukemia (CLL) cells but not on normal B-cells [6,9,54,55]. Deviant hematopoietic stem cells can give rise to monoclonal B-cells that have the capacity to self-renew and differentiate. In patients with CLL, 95% of these CSC-like neoplastic B-cells expressed elevated levels of ROR1 [6]. Another cohort saw a statistically significant increase in ROR1 levels after patients developed resistance to the BCL2 inhibitor venetoclax [54], offering a potential link between ROR1 and drug resistance in CLL. In transgenic mouse models of spontaneous CLL, ROR1 signaling is associated with an increase in tumor proliferation and CSC self-renewal as well as an activation of pAKT [56]. Targeted therapeutic strategies are essential to treating these CSCs and mitigating the increase in metastasis and relapse after treatment.

In MCL, an aggressive type of non-Hodgkin’s B-cell lymphoma, high ROR1 expression is linked to aggressive disease, reduced survival rates, and resistance to therapy [9]. ROR1 expression was highest in PDX models from patients who relapsed after treatment with brexucabtagene. Treatment of these resistant PDX models with VLS-101, a conjugate of monomethyl auristatin E to the humanized anti-ROR1 monoclonal antibody (mAb) UC-961, induced cellular cytotoxicity in ROR1-positive but not ROR1-negative cells in a dose-dependent manner [9]. Hence, in hematologic malignancies, the inhibition of ROR1 is sufficient to attenuate tumor growth, stressing the importance of understanding ROR1′s role in mediating treatment resistance.

## 5. ROR1 in Targeted Therapeutics and Clinical Trials

The restricted expression of ROR1 in normal adult tissues compared to its frequent overexpression in cancer, specifically in CSCs, makes it an attractive target for therapies aimed at eliminating both CSCs and differentiated cancer cells while minimizing off-target toxicity. Several therapeutic approaches targeting ROR1 are currently under investigation, showing promising results in preclinical and early clinical studies. Several mAbs targeting the extracellular domain of ROR1 have shown significant potential. Zilovertamab (formerly cirmtuzumab and UC-961), a humanized monoclonal antibody, has demonstrated the ability to inhibit WNT5A/ROR1 signaling in leukemic cells and was in clinical trials for CLL [6]. Zilovertamab has shown safety and encouraging signs of efficacy in patients with advanced breast cancer [49]. Antibody–drug conjugates (ADCs) combine the targeting ability of an antibody with the cytotoxic effect of chemotherapies. VLS-101, an ADC targeting ROR1, has shown effectiveness in preclinical models of MCL that are resistant to other therapies, including CAR T-cell therapy, indicating its potential to overcome acquired treatment resistance [9]. Another ADC, Zilovertamab vedotin was tested in patients with relapsing metastatic breast, pancreatic, lung, and ovarian cancers. This trial was halted for “business reasons” according to clinicaltrials.gov (NCT04504916); however, a recent publication reports that the drug was well tolerated despite being minimally effective in this patient population [57]. Small molecule inhibitors that target ROR1 are also under development. The ROR1 inhibitor Strictinin, an ellagitannin isolated from *Myrothamnus flabellifolius* [12], has demonstrated inhibitory effects on the spheroid-forming capacity of T98G glioblastoma stem-like cells, suggesting its potential in treating this aggressive brain cancer [34]. ARI-1, an inhibitor targeting the extracellular domain of ROR1, has shown promising results in preclinical models of chemoresistant non-small cell lung cancer [58]. KAN0439834, an inhibitor of the TK domain of ROR1, in combination with ibrutinib resulted in a significant increase in tumor death [59]. The inhibition of ROR1, through diverse treatment modalities, has emerged as a clinically promising modality across multiple cancers (Table 1).

Therapeutic regimens that combine ROR1-targeted agents with other anti-cancer treatments are also being investigated. Preclinical studies suggest that combining ROR1-targeted therapies with inhibitors of other oncogenic pathways can be more effective in eradicating drug-resistant cancer cells compared to single-agent treatments [21]. For example, combining cirmtuzumab with paclitaxel improved treatment efficacy in HER2-negative, metastatic breast cancer [49]. A combination of ibrutinib and zilovertamab was investigated in MCL before being withdrawn for “strategic reasons” (NCT05431179; clinicaltrials.gov). These findings emphasize how imperative the study of antibodies, small molecules and combination therapies is to achieving effective and durable ROR1-targeted treatments for improved patient outcomes.

## 6. Discussion

**Figure 2:**
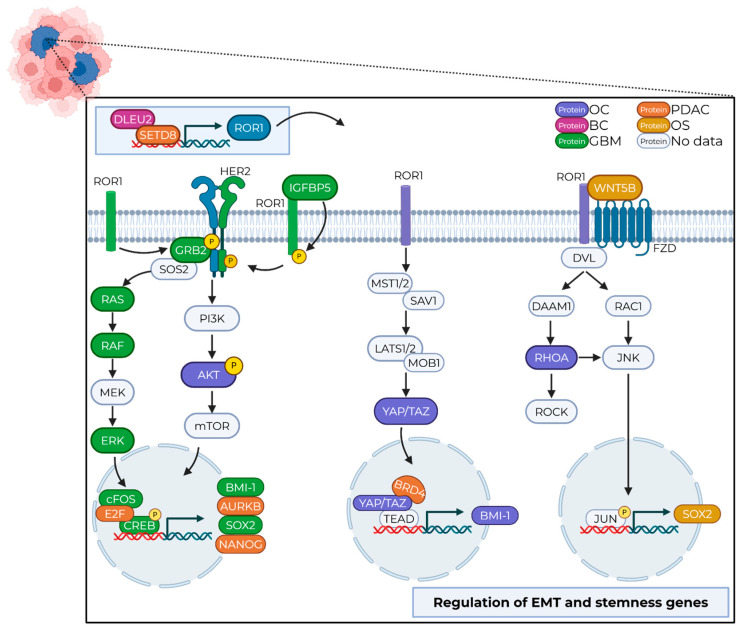
Summary of ROR1 signaling in CSCs. ROR1 crosstalk in CSCs depicting PI3K/RAS/AKT (left), YAP/TAZ (center), and WNT (right) signaling pathways. The PI3K/RAS/AKT pathway signals through receptor tyrosine kinases to regulate transcription, translation, cell survival, and proliferation [68]. The YAP/TAZ pathway promotes EMT, tumorigenesis, cell survival and migration/invasion [69,70,71,72], and the WNT pathway regulates immune control, metastasis, stemness, migration, and planar cell polarity [73]. Upper left tumor depicting bulk cells (pink) and CSCs (blue). Pathway components are defined as follows: Purple = OC, ovarian cancer; Pink = BC, breast cancer; Green = GBM, glioblastoma; Orange = PDAC, pancreatic cancer; Yellow = OS, osteosarcoma; Gray = No data shown in CSCs but is part of the established molecular signaling knowledge. Figure created in BioRender.com.

This review has discussed how ROR1 is upregulated in CSC tumor populations across numerous cancers and is responsible for maintaining stemness, driving aggressive disease progression and metastasis and serving not only as a marker of poor prognosis but also as a functional driver of adverse patient outcomes. While the signaling and clinical outcomes of ROR1 have been studied extensively in the total tumor population, its specific role in CSC biology remains poorly understood. Despite strong emerging evidence that ROR1 drives EMT and chemoresistance, there remains a critical gap in our understanding of how ROR1 orchestrates these processes within CSCs (Figure 2).

The aggressive and highly tumorigenic notoriety of CSCs makes this population of cells highly enticing for therapeutic targeting. Clinical trials targeting markers such as CD123, CD47, CXCR4 and others offer exciting progress in the field [43]. Even though there has been great advancement in recent years, the reality of CSC targeting is challenging due to the inherent nature of CSCs to enter quiescence, resist therapies and promote anti-apoptotic proteins, as well as the lack of specificity of many of the established CSC markers [43,44,74]. Moreover, there is still much about CSCs that we do not understand, including their interactions within the tumor microenvironment, their tumor-specific characterization, and complex epigenetic, transcriptional, and translational regulation [43].

One of the key challenges in targeting ROR1 lies in its complex and context-dependent signaling mechanisms. ROR1 functions as a signaling scaffold or co-receptor, forming heterodimers with other RTKs or FZDs as well as proteins such as HER2, CD19, or GRB2 to activate diverse downstream pathways. These pathways include WNT5A-RhoA, PI3K/AKT, YAP/BRD4/TAZ, STAT3, Notch, ERK1/2, and BMI-1, each of which plays a role in sustaining CSCs and driving disease progression. The upregulation of ROR1 by canonical WNT signaling [75] and YAP/BRD4 signaling [27] highlights positive feedback loops that could contribute to ROR1′s sustained expression. The frequent convergence of oncogenic signaling pathways around ROR1 underscores its importance in mediating intracellular crosstalk and extracellular cues from the tumor microenvironment to drive this malignant phenotype.

Effective ROR1 targeting requires elucidating the complex signaling interactions in isolated CSC populations and their impact on disease progression in different cancers. ROR1 targeting would likely be the most effective in co-treatment regimens by first inhibiting the CSCs to reduce drug resistance and metastasis, then following with debulking surgery and chemotherapies to eliminate the rest of the tumor [76]. Identifying additional ligands and co-receptors of ROR1 could provide further insights into its function and therapeutic vulnerabilities. Understanding the regulation of ROR1 by noncoding RNAs in CSCs is crucial for developing effective targeting strategies. Furthermore, understanding how ROR1 expression is modulated by the tumor microenvironment, such as immune infiltration, hypoxia, or mesenchymal stromal cells will be vital. Finally, investigating the potential of ROR1 as a diagnostic or prognostic biomarker in cancer could help identify patients who are most likely to benefit from ROR1-targeted therapies. Continued clinical trials evaluating the efficacy and safety of ROR1-targeted therapies in various cancers are essential to translate preclinical promise into clinical benefit. Still, it is likely that a single therapeutic will not be effective against the CSC population due to its relatively small number within the whole tumor. Therefore, the strength in targeting may lie in co-treatments with CSC-targeted therapy and whole tumor therapies, thereby disconnecting and dissolving the complex interplay between the CSCs, whole tumor, and tumor microenvironment.

ROR1 has emerged as a critical player in cancer biology, and its expression has recently been shown to be essential in CSC signaling. ROR1′s unique expression pattern, involvement in key oncogenic processes, and significant association with CSCs highlight its potential as a therapeutic target to overcome the limitations of conventional treatments. Understanding the specific signaling mechanisms in the context of CSCs is crucial for tailoring effective therapeutic interventions. Therefore, the next generation of ROR1 research in CSCs should approach the complex signaling pathways and decipher crosstalk between ROR1 and other stemness pathways. A multiomic approach integrating transcriptomics, immunoprecipitation proteomics, and spatial proteomics would provide a wealth of data to further our understanding of ROR1 in CSCs. Future research aimed at elucidating the intricate roles of ROR1 in CSC biology and its precise signaling mechanisms will pave the way for the development of innovative therapies that significantly improve outcomes for patients.

## Figures and Tables

**Table 1:** ROR1 Therapeutics in Clinical Development.

Therapeutic Class	Specific Therapeutic	Cancers in Trial	Clinical Status	References
mAb	Zilovertamab (Cirmtuzumab or UC-961)	B-ALL, DLBCL, neuroblastoma and Ewing sarcoma.	Phase 1; Recruiting	ClinicalTrials.gov ID NCT06395103
	Zilovertamab (Cirmtuzumab or UC-961) + Paclitaxel	HER2- BC	Phase 1b; Completed	[49]
Antibody–Drug Conjugate (ADC)	Zilovertamab (Cirmtuzumab or UC-961)	CLL	Preclinical, Phase 1, 2 (phase 1b-2 = cirmtuzumab + ibrutinib); Terminated (strategic directions)	[6,55,60]
	STRO-003	BC and LC	Preclinical	[61]
	VLS-101	TNBC, BC HER2- BC, NSCLC, GC, PDAC, and OC	Phase 2; Terminated (business reasons)	ClinicalTrials.gov ID NCT04504916; [57]
	VLS-101	MCL	Preclinical, Phase 1; Completed	[9,62]
Bispecific Antibody	Anti-ROR1 × CD3 BiAb	TNBC	Preclinical	[63]
CAR T-Cell Therapy	LYL-797	TNBC, NSCLC, OC, and EC	Phase 1; Active	ClinicalTrials.gov, NCT05274451
	ONCT-808	MCL, LBCL	Phase 1/2; Terminated (strategic directions)	[64]
	PRGN-3007	ROR1+ Hematologic and Solid Tumors	Preclinical, Phase 1/1b; Active	ClinicalTrials.gov, NCT05694364 [65]
	ROR1-CAR T	ALL, CLL, MCL, NSCLC, Osteosarcoma, PC, TNBC	Preclinical, Phase 1; Terminated (slow patient accrual)	ClinicalTrials.gov, (NCT02706392) [66,67]
Small Molecule Inhibitor	ARI-1	NSCLC	Preclinical	[58]
	KAN0439044	PDAC	Preclinical	[59]
	Strictinin	Glioblastoma	Preclinical	[9]

B-ALL = B-cell Acute Lymphoblastic Leukemia, DLBCL = Diffuse Large B-cell Lymphoma, LBCL = Large B-cell Lymphoma, BC = Breast Cancer, CLL = Chronic Lymphocytic Leukemia, TNBC = Triple-Negative Breast Cancer, LC = Lung Cancer, NSCLC = Non-Small-Cell Lung Cancer, GC = Gastric Cancer, PDAC = Pancreatic Ductal Adenocarcinoma, OC = Ovarian Cancer, EC = Endometrial Cancer, PC = Prostate Cancer.

## Data Availability

No new data was generated from this review.

## Abbreviations

The following abbreviations are used in this manuscript:

3′UTR	3′ untranslated region
ADCs	Antibody–drug conjugate
ALDH1	Aldehyde dehydrogenase 1
AURKB	Aurora kinase B
B-ALL	B-cell acute lymphoblastic leukemia
BC	Breast cancer
CLL	Chronic lymphocytic leukemia
CRD	Cysteine-rich domain
CSC	Cancer stem cell
DLBCL	Diffuse large B-cell lymphoma
EC	Endometrial cancer
EMT	Epithelial to mesenchymal transition
FZD	Frizzled
GSC	Glioblastoma stem cells
HIF1α	Hypoxia-inducible factor 1 alpha
Ig	Immunoglobulin
IGFBP5	Insulin-like growth factor-binding protein 5
KD	Kringle domain
LBCL	Large B-cell lymphoma
LC	Lung cancer
mAb	Monoclonal antibody
MCL	Mantle cell lymphoma
MSC	Mesenchymal stem cells
NICD	Notch intracellular domain
NSCLC	Non-small cell lung cancer
OC	Ovarian cancer
PC	Prostate cancer
PDAC	Pancreatic ductal adenocarcinoma
PDX	Patient-derived xenograft
PRD	Proline-rich domain
ROR1	Receptor tyrosine kinase-like orphan receptor 1
RTK	Receptor tyrosine kinase
T-DM1	Trastuzumab
TKD	Tyrosine kinase domain
TNBC	Triple-negative breast cancer
